# Disability trends among elderly Ukrainians in war conditions: a 10-year retrospective study

**DOI:** 10.1007/s40520-024-02863-y

**Published:** 2024-10-26

**Authors:** Alla Kyrychenko, Inna Khanyukova, Olena Moroz, Oksana Sirenko, Olexandr Kuryata

**Affiliations:** 1 State Institution, Ukrainian State Research Institute of Medical and Social Disability Problems of the Ministry of Health of Ukraine, Dnipro, Ukraine; 2 Department of Internal Medicine 2, Phthisiology, Occupational Diseases and Clinical Immunology of The Dnipro State Medical University, Dnipro, Ukraine

**Keywords:** Ukraine, War, Elderly, disability, Cardiovascular diseases

## Abstract

**Aim:**

Non-communicable diseases (NCDs) in elderly are a significant problem in Ukraine. It is expected that the ongoing war will augment this problem. The study aimed to analyze the trends of disability due to NCDs s in newly-diagnosed elderly patients between 2013 and 2023.

**Methods:**

This retrospective study included data obtained from the official document “Report on the Causes of Disability and Indications for Medical, Professional, and Social Rehabilitation” commissioned by the Ministry of Health of Ukraine. The data on disability due to NCDs s were obtained from for 2013–2023.

**Results:**

During the 2013–2021 the average number of disabled elderly patients due to NCDs per 10,000 elderly persons was significantly lower in compare with working-age. During the first year of full-scaled war the average number of disabled elderly patients due to NCDS s per 10,000 elderly persons dramatically increased by 2-fold, and in 2023 – by 2.8-fold against 2013 value (*p* < 0.05), while in working-age the disability rate increased significantly only in 2023 by 1.4-fold compared to the pre-war level. In 2013–2021 the main causes of disability among those related to NCDs in elderly were cardiovascular diseases, followed by cancer. In 2022 compared to the pre-war level, the indicators of disability of the elderly due to CVD increased by 1.5 times, due to cerebrovascular diseases - by 2.2 times, due to cancer - by 1.7 times, due to musculoskeletal diseases - by 2 times (*p* < 0.05). The most significant increase in the number of elderly people with disabilities during the full-scale war occurred due to coronary artery disease - by 55.1%, and osteoarthritis - by 83.4% from baseline in 2013. It estemated the differences in indicators of disability of elderly between regions of Ukraine, significantly higher indicators of disability of the elderly due to cardiovascular diseases, cancer, cerebrovascular diseases, musculoskeletal diseases were noted in the frontline regions.

**Conclusions:**

Data on primary disability among elderly Ukrainians indicates a significant increase in NCDs-related disability during the war in compare with working population, especially in frontline regions and regions with a high concentration of displaced persons. In this structure of NCDs-related increasing disability, CVD, oncology and musculoskeletal diseases system prevailed.

## Introduction

In accordance with the current data in the EU countries numbers of persons aged 65 + may increase from 84.6 million in 2008 to 151.5 million in 2060, while numbers of persons aged 80 + are projected to nearly triple, from 21.8 to 61.4 million [1, 2]. This rapid increase of the population above age 65 may result in a substantial increase in the future numbers of disabled persons in European countries [3].

Understanding trends in disability among older adults is critical to predicting and planning future health care costs and social benefits. Existing studies demonstrate the increase in life expectancy of the European population and the increase in the proportion of elderly people in the structure [4, 5].

In Ukraine, there are about 9 million elderly people - every fifth city dweller and every third village dweller [6]. The situation is described as the oldest humanitarian crisis in the world, where 24% of the population belongs to the age group over 60 years old [7]. 24% of elderly in Ukraine (about three million people) have a disability, compared to 13% on average of the entire population [7]. 20% of elderly people in Ukraine have physical impairments that affect their mobility.

The Russian invasion of Ukraine has had a devastating effect on civilians of all ages. In 2022, the number of internally displaced families increased 6 times [8]. Currently, the number of officially registered internally displaced persons in the country reaches 4.9 million people. Excessive population concentration in relatively safe regions causes problems with the availability and quality of basic services, in particular social and medical. On the other hand, for more than 2 years of full-scale war in Ukraine, 1,523 medical facilities were damaged and another 195 were completely destroyed [9]. As a result of enemy shelling, the medical institutions of Kharkiv, Donetsk, Mykolaiv, Kyiv, Chernihiv, Dnipro, Kherson and Zaporizhzhia suffered the greatest losses.

There is a particular threat to the elderly due to the vulnerability of this population category. Health status and psychosocial consequences wars for the elderly are sustainable and exciting. 11% of all displaced persons in Ukraine are elderly people, and the total number displaced persons reach 18% [8]. Thus, the elderly can stay more often in hard-to-reach places and in difficult conditions. These circumstances definitely have an impact on the rapid deterioration of the health of the elderly in wartime conditions.

This study aimed to analyze data on disability due to non-communicable diseases (NCDs) in newly-diagnosed patients in Ukraine during the full-scale war (2022–2023) and to compare these data with indicators of disability during Covid-19 pandemic period (2020–2021) and the prior year without war and pandemic conditions (2013).

### Participants and methods

#### Study design

This was a retrospective study.

#### Collected data

The analyzed data were obtained from the “Report on the Causes of Disability and Indications for Medical, Professional, and Social Rehabilitation” (Form No. 14) commissioned by the Ministry of Health of Ukraine. The form is filled out from the regional centers (bureaus) of the Medical and Social Expert Commission (MSEC) and submitted to the Ministry of Health. The analysis comprises all the data collected in Ukraine during the observed period except for occupied territories.

The data were analyzed for the period from 2013 to 2023. Russia’s full-scale invasion of Ukraine began on 24 February 2022, so the data collected in 2022–2023 are considered the data obtained during wartime.

Data were collected and presented according to the administrative division of Ukraine. The selection of specific regions for analysis was based on the following criteria: (1) the approach and impact of hostilities (2) the availability of medical care, the nature of the destruction of the network of medical institutions (3) the remoteness from the area of ​​hostilities and the presence of a significant concentration of displaced persons (4) proximity to the borders of the EU with the expected external migration. When analyzing regional indicators, they were divided into 3 groups: 1 group representatives of frontline regions (Zaporizhzhya, Kharkiv), 2 group - with a significant concentration of displaced persons (Dnipro, Odesa, Lviv), 3 group - distant from the front line and bordering EU countries (Zakarpattya, Volyn, Vinnytsia).

The diseases included in the study were classified according to the latest version of the International Classification of Diseases (ICD-11) [10]. The concept of disability was defined according to the biopsychosocial model according to the International Classification of Functioning, Limitations of Vital Activities and Health, which was approved in Ukraine in 2022 [11].

The study was conducted according to bioethics principles outlined in the Declaration of Helsinki, “Ethical Principles of Medical Research Involving Human Subjects”, the “Universal Declaration of Bioethics and Human Rights (UNESCO)” and approved by the Bioethics Commission of Ukrainian State Research Institute of Medical and Social Problems of Disability, Ministry of Health of Ukraine.

#### Data analysis

The data collected were analyzed as a part of the regular annual report of the Ukrainian State Research Institute of Medical and Social Problems of Disability. We used descriptive statistics while data processing, calculations were carried out using MS Excel for Windows (Microsoft Inc., Redmond, WA, USA). Analysis of variances (ANOVA) followed by post-hoc Tukey’s Multiple Comparison was used to verify significant difference between group means. *P* value of less than 0.05 was considered significant.

## Results

**Fig. 1:**
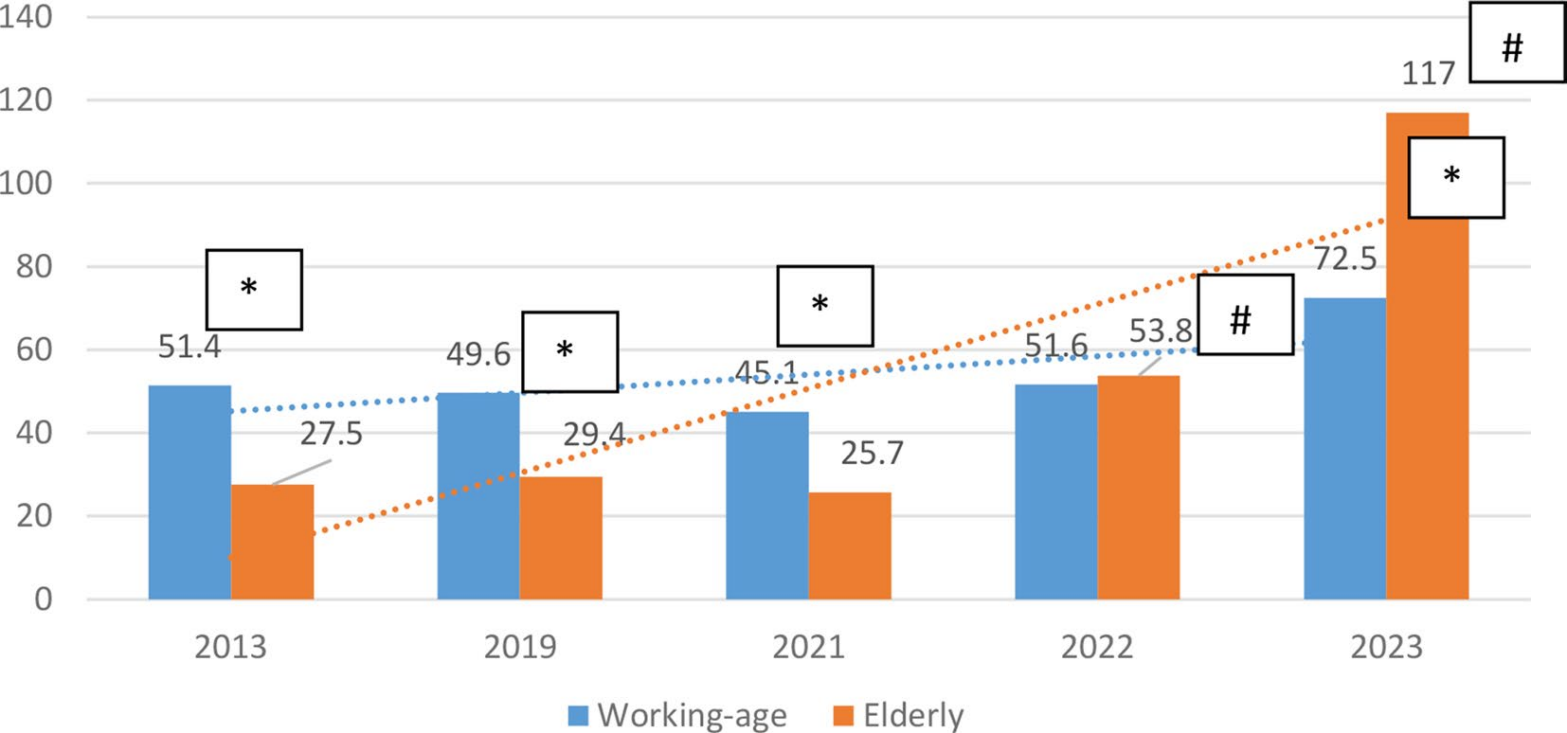
Dynamics of changes in indicators of disability in working-age and elderly per 10,000 elderly from 2013 to 2023 * - *p* < 0.05 the significance of the differences between the groups. #- *p* < 0.05 the significance of the differences within groups according to 2013 level

During the 2013–2021 the average number of disabled elderly patients due to NCDs per 10,000 elderly persons was significantly lower in compare with working-age (Fig. 1). During the 2022 the average number of disabled elderly patients due to NCDs per 10,000 elderly persons dramatically increased by 2-fold, and in 2023 – by 2.8-fold against 2013 value (*p* < 0.05), while in working-age the disability rate increased significantly only in 2023 by 1.4-fold compared to the pre-war level. (Fig. 1). In 2023, the level of disability among the elderly reached a significantly higher level compared to the working population on 38% (*p* < 0.05).

In 2013 the main causes of disability among those related to NCDs in elderly were cardiovascular diseases (average values per 10000 were 32) followed by cancer (26 per 10000). Cerebrovascular disease, psychiatric diseases and musculoskeletal diseases accounted for 4.7, 11.2 and 15.4 per 10,000 persons in the elderly population. In 2021 the main causes of disability were still cardiovascular diseases (average values per 10000 were 28.1) followed by cancer (26.2 per 10000). Cerebrovascular disease, psychiatric diseases and musculoskeletal diseases accounted for 12.8 (*p* < 0.05 in compare with 2013) and 16.2 per 10,000 persons in the elderly population.

**Fig. 2:**
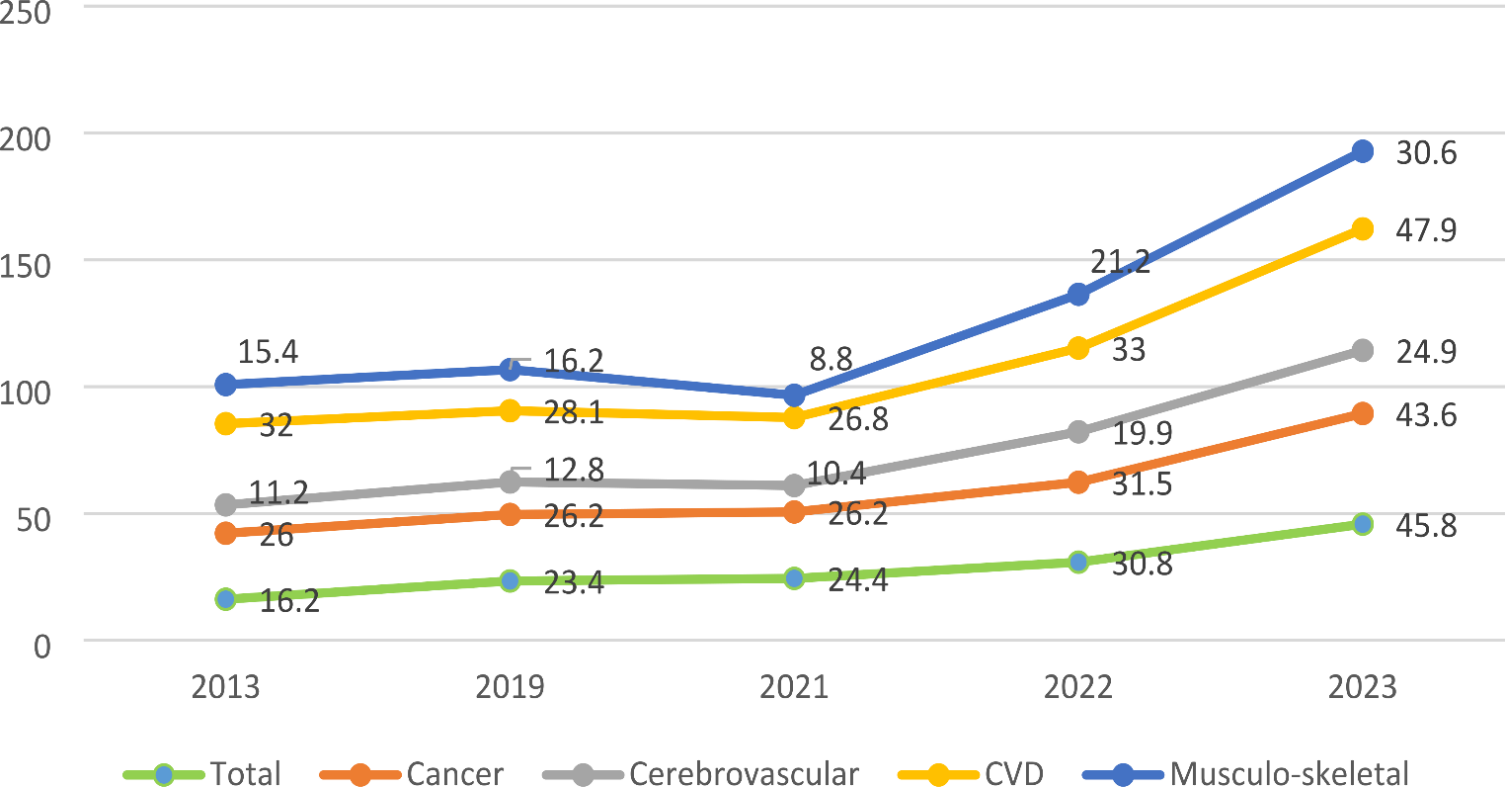
Dynamics of changes in indicators of disability due to main groups of NCDs per 10,000 elderly from 2013 to 2023

In 2022 compared to the pre-war level, the indicators of disability of the elderly due to CVD increased by 1.5 times, due to cerebrovascular diseases - by 2.2 times, due to cancer - by 1.7 times, due to musculoskeletal diseases - by 2 times (*p* < 0.05) (Fig. 2).

**Fig. 3:**
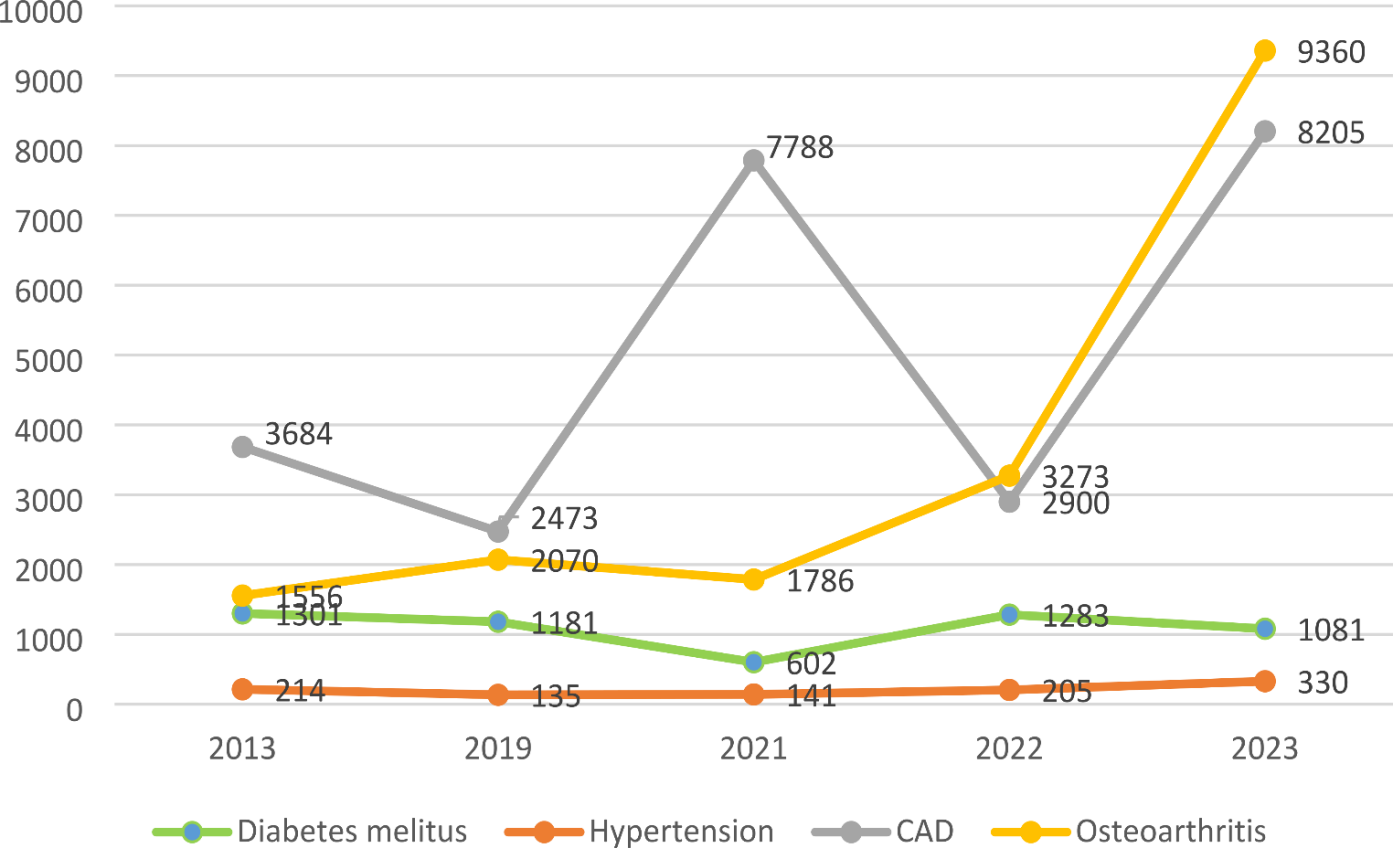
Dynamics of changes in total amount of elderly disability due to different nosologies of NCDs from 2013 to 2023

The most significant increase in the number of elderly people with disabilities during the full-scale war occurred due to coronary artery disease - by 55.1%, and osteoarthritis - by 83.4% from baseline in 2013 (Fig. 3). On the contrary, the number of disabled elderly people with diabetes decreased by 17% to the 2023 (Fig. 3).

The variability of the trend pattern for CAD was a result of two peaks (2021 and 2023) and decline (2022).

It should be noted the differences in indicators between regions of Ukraine. Therefore, significantly lower indicators of disability of the elderly due to cardiovascular diseases were noted in the 3^d^ region group (Zakarpattya) and 2nd region group (Lviv), significantly higher than the national average - in the 1st region group (Zaporizhzhia) (*p* < 0.05) (Table 1). During the 2013 in the 1st region group (Zakarpattya), rates of disability in the elderly due to cancer, cerebrovascular diseases, psychiatric diseases, and pathology of the musculoskeletal system are also significantly lower than the national average (*p* < 0.05) (Table 1). There was a significant increase in disability indicators in the regions of the 1st group (Zakarpattya) during the period 2013–2019, while the indicators decreased in the other regions (Table 1). There was still significant increase in 2021 disability indicators on NCDs in the 3^d^ region group (Zakarpattya) in compare with 2013, but slightly decreased compared to 2019.

**Table 1:** The average number of disabled elderly patients due to main groups of NCDs per 10,000 elderly persons in different regions of Ukraine in 2013–2023

Region	Cancer					Cerebrovascular disease, psychiatric diseases					Cardiovascular diseases					Musculoskeletal diseases				
	2013	2019	2021	2022	2023	2013	2019	2021	2022	2023	2013	2019	2021	2022	2023	2013	2019	2021	2022	2023
1st regional group (front-line regions)																				
Zaporizhzhya	23.7	33.2#	31.7#	46.6*#	63.5*#	2.1*	35.5#	25#	44.9*#	63.1*#	42.2*	16.9#	40.8*	44.9*	56.8*#	4.2*	28.7#	13.9#	20.2#	32.3#
Kharkiv	24.3	22.7	21.6	31.2#	46.2#	5.7	3.7	3.3	14.4#	8.7#	20.6	22.2	20.5	22.3	31.4#	9.4	10.3	4.6#	13*#	17.6*#
2nd regional group (regions with a large concentration of displaced persons)																				
Dnipro	23	22.6	24	31.8#	37.4#	6.2	7.3	7.9	15.4#	22.8#	28.6	29.6	30.7*	39.2#	45.2#	10.5	15.1	7.8	21.3#	30.3#
Odesa	24	27.7	32.6#	30.9	41.3#	4.6*	5.6	8.6#	20#	24.9#	32.2	28	23#	30.8#	38#	16.6	16.1	9.4	21.4#	29.6#
Lviv	27,1	19#	18.3#	27.1*#	43.8#	6,8	5.8	9.2	21.4#	26.5#	18,2*	14.9	16.6	28#	45.2#	9,7	6.2	5.5	11.9*#	23.7#
3^d^ regional group (regions distant from the front line)																				
Zakarpattya	10,1*	32.9#	36.1#	31.8#	45.1#	2,1*	13.3#	8.7#	16.1#	24.7#	8,1*	17.5#	22.3#	34.4	57.6*#	4,2*	25#	13.4#	23.7#	44.6*#
Volyn	25,2	21.6	21.6	42.4*	51.1*#	9,8*	7.5	15.1#	17.5#	17.9#	19,2	22.1	23.5	31.7#	34.6#	10,5	7.6	4.5#	23.7#	25#
Vinnytsia	34.5	23.3#	19.5#	28.4	43.3#	19.7*	12.3	9.2#	20	17	41.8*	26.9#	27.5#	29.1	50.1#	28.2*	11.9#	4.9#	11.6*#	22.6#

*- the significance of the differences in comparison with the average indicator for Ukraine

# - the significance of the differences in comparison with 2013

Reliable differences in the prevalence of nosologies among the elderly before the full-scale war were established depending on the geographical location in Ukraine (Table 1). Therefore, the 1st group regions (Dnipro, Kharkiv) were characterized by a significantly higher number of elderly patients with diabetes mellitus, osteoarthritis compared to the national average in 2013 (*p* < 0.05). The regions distant from the 3^d^ group (Zakarpattya, Volyn) were characterized by a significantly lower number of elderly patients with diabetes mellitus, coronary artery disease, osteoarthritis compared to the national average in 2013 (*p* < 0.05). During the period 2019–2021, the indicators of disability due to coronary artery disease increased sharply in almost all regions of Ukraine and achieved a significant growth of more than twofold in total (Table 1). These changes are in trends may have been a consequence of the Covid-19 pandemic. During the period from 2013 to 2019, there was a reduction in the total number of elderly people with disabilities due to cardiovascular diseases, diabetes, hypertension without differences by region, which may indicate this reduction due to the occupation of part of Ukraine. However, despite these trends, a significant increase in disability due to osteoarthritis has been noted (*p* < 0.05), this trend continued in 2021.

Analysis of indicators of disability of the elderly during the period of full-scale war, depending on the region of Ukraine, revealed a significant increase in indicators in almost all groups of NCDs among all of regions (*p* < 0.05) (Table 2). The most noticeable increase in disability rates was in the 1st group (Zaporizhzhia): cancer - by 53.7%, cerebrovascular diseases - by 62.8%, CVD - by 43.7%, diseases of the musculoskeletal system - by 33.5% (*p* < 0.05) (Table 2). The analysis of the number of cases of elderly disability from different nosological forms depending on the region of Ukraine demonstrates a significant decrease in indicators at the beginning of a full-scale war and an almost twofold increase in the number of cases in 2023 in compare with 2021 among almost all regions of Ukraine (Table 2). Therefore, the 2nd and 3^d^ groups (Lviv, Zakarpattya) were characterized by a significantly higher number of elderly patients with CAD, diabetes mellitus, osteoarthritis compared to the national average and 2021 (*p* < 0.05).

**Table 2:** The number of disabled elderly patients due to main of NCDs nosology in 2013–2021

Region	diabetes mellitus					arterial hypertension					coronary artery disease					osteoarthritis				
	^2013^	^2019^	^2021^	^2022^	^2023^	^2013^	^2019^	^2021^	^2022^	^2023^	^2013^	^2019^	^2021^	^2022^	^2023^	^2013^	^2019^	^2021^	^2022^	^2023^
1st regional group (front-line regions)																				
Dnipro	107*	70#	63#	112*#	159#	13	7	9	17*#	8	144	161	562#	193#	320#	121*	175	135	295*	605*#
Zaporizhzhya	45	42	34	64#	126#	12	13	15	7#	51*#	160	171	648#	186#	461#	83	242#	209#	222	530#
2nd regional group (regions with a large concentration of displaced persons)																				
Kharkiv	85*	60	56	56	145#	12	0	0#	1	14	185	200	424#	195#	496#	52	95	83	110*	444#
Odesa	64	85	65	126*#	263*#	18	25	29	45*#	67*#	190	170	532#	884*#	564*#	57	87	65	239	544#
Vinnytsia	78	59	48#	65	173#	22	7	8#	25*#	43*	269*	102#	331	160#	621*#	141*	83#	96	188	1005*#
3^d^ regional group (regions distant from the front line)																				
Lviv	58	56	59	114*#	297*#	0	0	0	0	1	158	147	419#	281*#	1033*#	85	77	76	220	678*#
Zakarpattya	34*	45	54#	114*#	176#	6	3	3	5	171*#	15*	36#	180#	171	300#	26*	63	158#	212	288*
Volyn	9*	16*	17#	19*	50*#	2	1	1	4	4	42*	65	158#	73#	220*#	20*	18	29	34	135*#
Average	61	57.5	27.5#	66.5#	135#	12	5	5.5#	6	28.5	159	154	421.5#	189.5#	478.5	70	85	89.5	216#	537#

*- the significance of the differences in comparison with the average indicator for Ukraine

# - the significance of the differences in comparison with 2013

## Discussion

NCDs s place a significant social and economic burden, especially in middle-income countries [12]. Here we showed that, as in recent decades, in war period of 2014–2023, the NCDs s continued to play a leading role in disability in Ukraine, especially in elderly population. Furthermore, in the presence of a full-scale war, the specific weight of NCDs-related disability among elderly people is increasing more pronounced. Massive stress influence, destruction of health care systems, paralysis of the economy, anxiety, and/or depression contribute to the progression of NCDs and the development of complications.

This is especially important for CVDs, which showed a steep upward trend during the whole observed period. According to the SYNTAX Study, findings suggest that exposure to multiple war-related stressors may increase the complexity and severity of CAD in Syrian war survivors [13].

The observed increase in CVD between 2021 and the first years of the war (2022–2023) are in line with previously published articles [14, 15]. Thus, the most common cause of visit to doctors in front-line regions was cardiovascular diseases (17.9%), while the frequency of non-respiratory infections remained stable.

In a systematic review conducted by Mahase E. et al., it was found that living in a war zone are directly linked to an increased risk of CAD, stroke, diabetes, hypertension, as well as increased cigarette and alcohol consumption [16]. However, the studies included in the systematic review did not provide sufficient detail to understand the causal relationship between armed conflict and CVD [16]. The explanation for the observed link between war and NCDs is likely to be complex and multifactorial, possibly driven by a stress response besides changes in health behaviors at the individual level and disruptions to healthcare provision at the population level.

Based on the results of this study, the regions of Ukraine are characterized by a significant regional variation of morbidity from NCDs, with the concentration of its higher levels in the front-line regions and regions with significant flow of internally displaced persons.

The established trend of CVD during the analyzed period revealed two peaks (2021 and 2023) of growth and a peak of decline (2022) in the number of elderly disabled people. The growth peak in 2021 could be due to the Covid-19 pandemic, while the decline in 2022 could be due to deaths among the elderly due to hostilities and evacuations, which were most intense in the first year of full-scale war. The subsequent peak of growth in the number of elderly disabled people in 2023 can be explained as a result of the impact of military factors on the state of CVD.

This study had several limitations. First, our results were observational, so it is impossible to establish a causal link between the first year of war and observed changes in the incidence of NCDs. Second, the proper trend analysis requires longer observational periods, and third, our database is missing data from occupied or partially occupied regions of Ukraine. The observed increase threatens permanently disability of the country’s elderly population, which determines the priority of further improvement of primary, specialized medical, and social care in wartime conditions. The current situation following Russian aggression on Ukraine requires a comprehensive and regionally differentiated approach by the Ukrainian government and agencies in reducing morbidity and disability in elderly due to CVDs, cancer, musculoskeletal diseases. Postconflict reconstruction efforts should aim to deliver low-resource preventative interventions through primary care to prevent threatening excess morbidity and mortality.

## Conclusion

Our data on primary disability among elderly Ukrainians indicates a significant increase in NCDs-related disability during the war in compare with working population, especially in frontline regions and regions with a high concentration of displaced persons. In this structure of NCDs-related increasing disability, CVD, oncology and musculoskeletal diseases system prevailed. The observed trends determine the priority of further improvement of primary, specialized cardiac and medical, and social care in wartime conditions.

## Data Availability

No datasets were generated or analysed during the current study.
